# Important prognostic factors for survival in patients with malignant pleural effusion

**DOI:** 10.1186/s12890-015-0025-z

**Published:** 2015-03-28

**Authors:** Mauro Musa Zamboni, Cyro Teixeira da Silva, Rodrigo Baretta, Edson Toscano Cunha, Gilberto Perez Cardoso

**Affiliations:** Pulmonology and Thoracic Surgery Division, Hospital do Câncer I, Instituto Nacional de Câncer/Ministério da Saúde, Rio de Janeiro, RJ Brazil; Pulmonology Division, Hospital Antonio Pedro, Universidade Federal Fluminense, Niterói, RJ Brazil

**Keywords:** Neoplasm, Malignant pleural effusion, Prognosis, Analysis, Survival

## Abstract

**Background:**

The approach to palliative treatment of malignant pleural effusion (MPE) should be individualized because these patients generally have poor survival. Our study aimed to develop a model to identify prognostic factors or survival time in patients diagnosed with MPE.

**Methods:**

This is a retrospective, descriptive, observational study to identify prognostic factors related to MPE in patients with a confirmed cancer diagnosis. Cox regression analysis was used to determine significant potential prognostic factors with respect to survival time. Survival time was defined as the time from pathological diagnosis to death.

**Results:**

One hundred and sixty-five patients were included; 77 were men (47%) and 88 were women (53%). The median age was 60 years, and all of the patients were pathologically proven to have MPE. Non-small-cell lung cancer (36.0%), breast carcinoma (26%), and lymphoma (13.0%) were the most frequently diagnosed tumors. The median overall survival of patients from the initial diagnosis was 5 months (range: 1.0–96.0 months). Kaplan–Meier univariate analysis showed that survival was significantly related to the following prognostic factors: ECOG PS (hazard ratio [HR] 10.0, 95% confidence interval [95% CI] 5.96 to 18.50, p < 0.0001), primary cancer site (HR 1.99, 95% CI 1.23 to 3.22, p < 0.01), positive pleural cytology (HR 1.25, 95% CI 0.88 to 1.78, p = 0.04), and positive histology (HR 1.33, 95% CI 0.97 to 1.81, p = 0.04). Other potential independent diagnostic factors that were examined did not affect survival. Cox regression analysis showed that only the ECOG PS was highly predictive of survival (HR 73.58, 95% CI 23.44 to 230.95, p < 0.0001).

**Conclusions:**

ECOG PS is an independent predictor of survival in patients with MPE at initial diagnosis. This prognostic factor can help physicians select patients for appropriate palliative treatment of this syndrome.

## Background

A malignant pleural effusion (MPE) is often the first sign of cancer and it is a prognostic factor in patients with advanced disease. MPE can be a complication of any malignancy, but in patients with lung cancer, the frequency of MPE ranges from 7% to 23% [1] MPE is characteristic of advanced malignancies, but it may also appear in patients with a longer projected survival (e.g., those with lymphomas, including Hodgkin’s disease, and breast carcinoma). The quality of life in patients with MPE is usually compromised because of distressing symptoms, such as coughing, dyspnea, and chest pain [2-4].

The presence of MPE signifies an advanced stage of disease and usually indicates that death will likely result within a few months of the time pleural fluid is first detected [4,5]. Several treatments can relieve the respiratory symptoms of MPE. If the expected survival is short, less-invasive procedures are preferred for MPE [5-8].

Considering the cost of treatment for MPE and its potential complications, there are limited data that might assist chest physicians or surgeons in the precise prediction of survival time for patients with MPEs [7]. In this study, we investigated different variables that are potentially correlated with prognosis in a group of patients with MPE at the time of diagnosis [9-12]. This study aimed to determine the relative contributions of each prognostic factor with respect to the survival time of patients with MPE.

## Methods

A retrospective study was designed to identify prognostic factors in patients with MPE and a confirmed diagnosis of cancer. It was conducted from 2010 to 2012 at the Instituto Nacional do Cancer (INCA), Rio de Janeiro, Brazil. Data were collected from the medical records of patients who were identified through the cancer registry. One hundred and sixty-five patients with MPE who were referred to the hospital were included in this study. The Ethics Committee of INCA do Cancer, Rio de Janeiro, Brazil, approved this study in accordance with the recommendations found in the Declaration of Helsinki (#162930; Jan 14, 2013).

At the INCA, detailed historical background was analyzed, physical examinations were conducted, and imaging evaluation was performed for each patient with clinical manifestations compatible with MPE. The presence of pulmonary or pleural masses, pulmonary atelectasis, or lymphadenopathy on chest radiography or/and computed tomography was considered suggestive of malignancy [5].

In addition, thoracocentesis was performed using standard methods. A pleural biopsy was performed using a Cope’s needle and/or video-assisted thoracoscopic surgery. The definitions used for the diagnosis of a pleural effusion were based on previously published criteria [5]. When the diagnosis was unclear after thoracocentesis or closed-needle pleural biopsy, when the effusion persisted and symptoms increased, or when malignancy could not be differentiated from tuberculosis, the patient was referred for thoracoscopy or thoracotomy [5].

In all cases, the diagnosis of MPE was established by the presence of malignant cells in the pleural fluid upon thoracocentesis (positive pleural cytology) or evidence of a neoplasm upon pleural biopsy (histologically) [5].

The inclusion criteria for the study consisted of all patients with MPE who were not submitted to specific procedures, such as pleurodesis, pleuroscopy, or thoracoscopy. The exclusion criteria consisted of previous chemical pleurodesis and undiagnosed pleural effusion.

### Potential predictors of survival

We considered 12 potential independent prognostic factors for survival in 165 patients with MPE from the INCA database. The database included demographic characteristics (age and sex), primary tumor site, glucose in the pleural fluid, levels of total protein and lactate dehydrogenase in the pleural fluid, cytological and histological results, percentage of lymphocytes and neutrophils in the pleural fluid, biochemical classification of pleural fluids into transudates or exudates [5,6], and Eastern Cooperative Oncology Group Performance Status (ECOG PS). Briefly, ECOG PS consists of 5 grades: normal activity, grade 0; symptomatic but fully ambulatory, grade 1; symptomatic but bedridden less than 50% of the time, grade 2; bedridden more than 50% during the daytime, grade 3; completely (100%) bedridden, grade 4; and dead, grade 5 [13-15] (Table 1). The survival time (measured in months) was defined and calculated from the day of pathological diagnosis to the day of death. No patient was censored.

**Table 1:** **Performance scales: ECOG scores [** 14 **,** 15 **]**

**ECOG grade**	**ECOG status**
0	Fully active, able to carry on all pre-disease performance without restriction
1	Restricted in physically strenuous activity but ambulatory and able to carry out work of a light or sedentary nature, e.g., light house work, office work
2	Ambulatory and capable of all selfcare but unable to carry out any work activities up and about more than 50% of waking hours
3	Capable of only limited selfcare, confined to bed or chair more than 50% of waking hours
4	Completely disabled. Cannot carry on any selfcare. Totally confined to bed or chair
5	Dead

### Statistical analysis

Statistical analyses were performed using the MedCalc software, version 13.2.2, (Mariakerke, Belgium) [16]. Categorical variables are expressed as ratios of the two values (percentages). Continuous variables that were not normally distributed are expressed as medians after performing the D’Agostino–Pearson test.

The relationship between prognostic factors and outcome was modeled statistically by univariate Kaplan–Meier survival analysis [17]. For each potential predictor, stepwise modeling was performed to screen variables for inclusion into the model. A p value less than or equal to 0.10 by the chi-square test was required for a potential predictor to enter in the model. The multivariate Cox regression or proportional hazards regression method was used for investigating the effect of several independent variables (prognostic factors) for survival-time. The Cox model provides an estimate of the hazard ratio and its 95% confidence intervals (CIs). The resultant risk variables in the Cox regression analysis were visualized by Kaplan–Meier curves. Statistical comparisons were performed using Kaplan-Meier method with log-rank test. A type I error probability of 0.05 (a two-tailed p-value) was used as the threshold for statistical significance. A 95% CI was calculated to assess the clinical importance of the results.

The sample size that was necessary for this study was determined based on a publication by Altman and Royston [17]. Survival analysis with the Kaplan–Meier method [18] can be used to study any sample size but is especially useful in studies with a small number of observations. In a regression model, the number of events should be at least 10 times the number of potential prognostic variables [19-21]. According to Royston and Altman [22] a prognostic model should not enter in clinical practice unless it demonstrates that it performs a useful role. Our statistical approach to external validation of a Cox model included: appropriate sample size, a multivariable model and its coefficients, creation of risk groups and Kaplan–Meier curves [19-22].

## Results

Pleural effusion was the first manifestation of malignancy in approximately 15% of asymptomatic patients. The most common symptoms that were reported by patients were dyspnea (80%) with a modified Medical Research Council (mMRC) score of 2 (moderate) to 4 (very severe), dull chest pain (30%), and nonproductive cough (10%). In 40% of patients with a personal history of cancer and with chronic symptoms of disease (longer than 30 days), many (90%) patients had symptoms attributable to the cancer itself, such as fever, anorexia, weight loss, and malaise. A total of 52% of pleural effusions were large (affected two thirds or more of the hemithorax) and 33% were massive (opacified the entire hemithorax).

**Figure 1:**
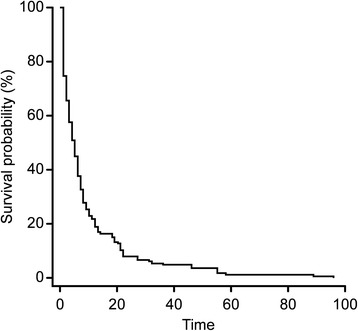
**Kaplan-Meier curve showing the survival of the 165 patients.** The median survival for all patients in the group was five months (95% CI; range: 1.0-96.0). (time in months).

Table 1 shows the ECOG PS scores. Table 2 shows the characteristics of the 165 patients in the study population and the causes of MPE. Table 3 and Figure 1 show survival analysis according to the type of primary tumor. All of our patients had a median survival of 5 months (range: 1.0 – 96.0 months). Survival time was calculated in months rather than in days because it is a classical time variable according to multiple authors [20,21]. Patients with MPE from ovarian cancer showed better survival than those whose cancers were located in other anatomical sites (Table 3). Table 4 shows that some prognostic factors (variables) were not available in all cases. Table 4 shows the Kaplan–Meier univariate analysis, which showed that survival was significantly related to ECOG PS (chi-square = 195.40, p < 0.0001), the site of the cancer (chi-square = 5.54, p < 0.01), pleural cytology (chi-square = 4.20, p = 0.04), and histology (chi-square = 4.09, p = 0.04). With regard to the cancer site, only ovary tumors were significant compared with other malignant tumors. Other potential independent diagnostic factors that were examined did not appear to affect survival time.

**Table 2:** **Baseline characteristics of the study population (n = 165)**

**Characteristics**	Values*
Males, n (%)	77.0 (47.0)
Females, n (%)	88.00 (53.0)
Median age (range), years	60.0 (1.0-95.0)
Median pleural fluid glucose level (range), mg/dL	96.0 (2.0-440.0)
Median pleural fluid LDH level (range), U/L	589.0 (124.0-5506.0)
Median pleural fluid proteins (range), g/dL	4.4 (0.5-5.9)
Median pleural fluid neutrophils level (range), %	13.0 (0.0-99.0)
Median pleural fluid lymphocytes level (range), %	75.0 (1.0-100.0)
ECOG PS, grade 0 (%)	9 (5.0)
ECOG PS, grade 1 (%)	9 (5.0)
ECOG PS, grade 2 (%)	19 (12.0)
ECOG PS, grade 3 (%)	47 (29.0)
ECOG PS, grade 4 (%)	81 (49.0)
Positive pleural cytology, n (%)	89 (54.0)
Positive pleural histology,, n (%)	83 (50.0)
Exudate, n (%)	70.0 (92.0)

*Missing data: ninety patients had missing data for glucose and protein levels; ninety three for LDH; one hundred sixteen for neutrophils; one hundred seventeen for lymphocytes and eighty nine for biochemical classification of pleural fluids. LDH: lactate dehydrogenase; PS: performance status.

**Table 3:** **Survival analysis according to primary tumor type**

**Type of primary tumor**	**Patients, number (%)**	**Median survival time, months (range)**
Ovary	10 (6.0)	21.0 (5.0-46.0)
Breast	43 (26.0)	6.0 (1.0-58.0)
Lymphoma	21 (13.0)	4.0 (1.0-55.0)
Lung	59 (36.0)	4.0 (1.0-96.0)
Unknown	08 (5.0)	4.0 (1.0-13.0)
Other	24 (14.0)	3.5 (1.0-89.0)
Overall	165 (100.0)	5.0 (1.0-96.0)

**Table 4:** **Univariate analysis of the association between potential prognostic factors and survival for all 165 patients with malignant pleural effusions**

**Prognostic factors**	**Categories**	**Patients (n)**	**Median survival time (months) – (95% CI)**	**Hazard ratio (95% CI)**	**P value**
ECOG performance status	0	9	55.0 (46.0-58.0)	1.0	< 0.0001*
Status	1	9	22.0 (22.0-27.0)	1.67 (0.93-3.01)	
	2	19	18.0 (13.0-19.0)	2.13 (1.24-3.63)	
	3	47	7.0 (6.0-8.0)	3.72 (2.26-6.11)	
	4	81	1.0 (1.0-2.0)	10.50 (5.96-18.50)	
Gender	Male	77	3.0 (2.0-5.0)	1.14 (0.80-1.61)	
	Female	88	6.0 (4.0-7.0)	1.0	0.4105
Pleural fluid	<13.0	24	4.0 (2.0-6.0)	1.0	
Neutrophils (%)	≥13.0	25	4.0 (2.0-8.0)	1.23 (0.70-2.16)	0.4091
Pleural fluid glucose (mg/dL)	<96.0	37	3.0 (2.0-7.0)	1.04 (0.66-1.64)	
	≥96.0	38	5.0 (3.0-6.0)	1.0	0.8323
Pleural fluid LDH (U/L)	<589.0	36	6.0 (3.0-8.0)	1.0	
	≥589.0	36	3.5 (2.0-7.0)	1.18 (0.74-1.88)	
Pleural fluid total	<75.5	24	4.5 (2.0-7.0)	1.0	0.4247
lymphocytes (%)	≥75.5	24	3.5 (2.0-7.0)	1.14 (0.64-2.01)	0.6040
Age (years)	<60	79	5.0 (3.0-7.0)	1.0	
	≥60	86	4.0 (3.0-6.0)	1.08 (0.79-1.47)	0.5618
Pleural fluid total	<4.4	36	5.0 (2.0-6.0)	1.0	
proteins (g%)	≥4.4	39	5.0 (3.0-7.0)	1.26 (0.80-2.00)	0.2590
Histopathology	Positive	83	6.0 (2.0-5.0)	1.33 (0.97-1.81)	
	Negative	82	4.0 (4.0-9.0)	1.0	0.0429*
Cytology	Positive	89	6.0 (4.0-8.0)	1.25 (0.88-1.78)	
	Negative	76	3.5 (2.0-5.0)	1.0	0.0403*
Classification	Exudate	70	4.0 (3.0-6.0)	1.04 (0.44-2.43)	
	Transudate	6	5.0 (1.0-8.0)	1.0	0.9161
Cancer site	Other	155	4.0 (3.0-6.0)	1.0	
	Ovary	10	21.0 (8.0-22.0)	1.99 (1.23-3.22)	0.0186*

*p value (unadjusted), Chi-square ≤ .10 = statistically significant variables; ECOG: Eastern Cooperative Oncology Group; LDH: lactate dehydrogenase; CI: Confidence Interval.

Patients with an ECOG PS grade of 0 had the longest median survival (55 months), while those with a grade of 1, 2, 3, or 4 had a median survival of 22, 18, 7, and 1 month, respectively (Table 4). Patients with pleural effusion and ovarian cancer had the best median survival (21 months) compared with those with other primary tumors. The medial survival of patients with breast cancer was 6 months, and those with either lung cancer or lymphoma had a median survival of 4 months (Tables 3 and 4).

**Figure 2:**
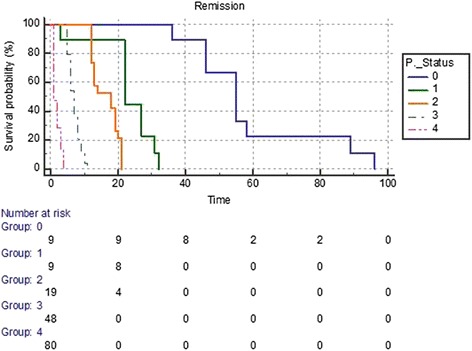
**Kaplan-Meier curves showing the relationship between ECOG performance status (PS) and survival in patients with MPEs.** A significant difference was observed in survival for patients with an ECOG grade of 1 to 4 (Chi-square = 242.15, p < 0.0001). ECOG scores of 1 (HR: 1.67, 95% CI: 0.93-3.01, p > .05), 2 (HR: 2.13, 95% CI: 1.24-3.67, p < .05), 3 (HR: 3.72, 95% CI: 2.26-6.11, p < .05) and 4 (HR: 10.50, 95% CI: 5.96-18.50, p < .05) are shown. (time in months).

Cox proportional hazards analysis showed that ECOG PS (HR 73.58, p < 0.0001) was the only independent prognostic factor affecting the survival of patients with MPE (Table 5). If the regression coefficient of the prognostic factor was positive, the risk of death (hazard) was higher (e.g., patients with higher values had a worse prognosis (Table 5). Patients with an ECOG score of 4 had worse survival than those with a better PS (ECOG score of 1 or 2), as shown by the Kaplan–Meier curve (Figure 2).

**Table 5:** **Cox proportional regression analysis for statistically significant prognostic factors by univariate analysis in relation to the survival of all 165 patients with malignant pleura effusions**

**Factors**	**Regression coefficient (b)**	**Hazard ratios**	**95% CI of HR**	P value (adjusted)*
ECOG PS	4.2984	73.58	23.44 – 230.95	<0.0001
Histopathology	0.3752	1.45	0.86 – 2.43	.15
Cytology	−0.1557	0.85	0.51 – 1.41	.54
Cancer site	0.053	1.05	0.52 – 2.14	.88

*Over model fit: Null model −2 log likelihood: 1387.549. Full model −2 log likelihood: 1075.968, Chi-square = 311.58, p < 0.0001; CI: confidence interval; ECOG: Eastern Cooperative Oncology Group.

In summary, we found that the ECOG performance was a predictor of survival in patients with MPE from Brazil (Table 5).

## Discussion

This retrospective study was designed to identify prognostic factors in patients with MPE and a confirmed diagnosis of cancer. A classical statistical model was appropriate to achieve this objective.

All of the patients in our study were diagnosed by conventional tests and procedures, and none received treatment for MPE (Table 2). The ratio of male to female subjects and the median age of 60 years are similar to the demographic characteristics as described by other authors in several series [23,24].

An isolated small-sized MPE is a prognostic factor that is associated with a significantly worse survival in patients with lung cancer [25,26]. However, this is dependent on the stage and histopathological classification of the malignancy. Similar to several studies mentioned by Light [7,8], lung carcinoma (36.0%), breast carcinoma (26.0%), and lymphoma (13.0%) were the most frequent primary diagnoses in our series (Table 2). All of our patients with MPE and lung cancer had non-small-cell lung cancer. However, in a study by Jimenez et al. [27], massive MPEs (unlike small MPEs) were associated with a worse survival, independent of age and histological group.

According to The International Staging System for Lung Cancer [28], survival time for patients with MPE is worse in all stages of lung cancer [29]. Currently, the anatomical extent of MPE, as determined by the TNM staging system, is the most important prognostic tool for lung cancer. In 2007, The International Association for Study of Lung Cancer established that the presence of pleural effusion results in the designation of T4 disease. The presence of MPE is considered as metastatic disease (M1b) [30]. The survival time following diagnosis ranged from 1 to 96 months (median: 5 months) in the patients in our study (Table 3). Sears and Hajdu [31] demonstrated identical results to our study, with an average survival of 5 months or less following the diagnosis of MPE. In our study, patients with ovarian cancer and MPE had a longer survival (21 months, range: 5.0–46 months) than those with cancers of other primary sites (Table 3). The most frequent extra-abdominal metastatic site in cases of ovarian carcinoma is the pleural cavity [32,33]. The median survival in a group of 214 patients with ovarian carcinoma and MPE (stage IV) was 24 months [34,35]. In a study by Anevlavis et al. [24], patients with lymphoma had the best median survival (26 months), and those with ovarian and breast carcinomas had the second best survival times (18 and 15 months, respectively). In 120 cases of MPE reported by Martinez-Morangon et al. [9], the overall survival was 9 months.

In the current study, Kaplan–Meier univariate analysis showed that survival was significantly related to ECOG PS, primary cancer site, positive pleural cytology, and positive histology. Other potential independent diagnostic factors that were examined had no effect on survival (Table 4). Among 171 patients who were included in a study by von de Molengraft and Vooijs [36], only 4% with positive pleural fluid cytology survived for 2 years after diagnosis.

In our study, Cox regression analysis demonstrated that only ECOG PS was highly predictive of survival. Several studies have demonstrated that performance status is a prognostic factor in cancer subjects [15,20-25]. The ECOG scale evaluates disease progression and quantifies the extent to which the disease affects the daily living abilities of the patient [15].

Our findings are in accordance with those of Burrows et al. [23] and Anevlavis et al. [24]. Both of them found that Performance Scale scores were predictive of survival in patients with MPE. Burrows et al. [23] found that only Karnofsky Performance Scale scores at the time of thoracoscopy were predictive of survival in patients with recurrent symptomatic MPE. Anevlavis et al. [24] concluded that prognostic factors affecting survival in patients with MPE were performance status (ECOG grade), primary tumor histology, and the neutrophil-to-lymphocyte ratio. Bielsa et al. [26] showed that tumor type and some biochemical features of pleural effusion (pH and concentrations of protein and lactate dehydrogenase) affect survival in patients with MPE.

Our findings – pH of pleural fluid and glucose levels - are different from those of several other authors. In a study by Heffner et al. [37], the pH of pleural fluid was not predictive of the need for pleurodesis in selected patients, based on estimated survival. However, Rodrigues-Panadero and Lopez-Mejias [38] concluded that pH (lower than 7.35) and glucose levels (lower than 60.0 mg/dL) in pleural fluid are risk factors for worse survival in patients with pleural cancer owing to extensive disease. Potential explanations for this discrepancy between different studies are the different regression models used for validation of the data, sample size, bias, and distinct populations used by other authors.

Our study adds important information to the existing literature. The projected survival time can help determine the most appropriate type of intrapleural therapy for MPE (e.g., pleurodesis vs. chronic drainage with indwelling catheters). There are several options for the treatment of MPE patients, some of which involve chemotherapy [5,8], including therapeutic pleural thoracentesis, chemical pleurodesis with intercostal tube drainage or after thoracoscopy, a pleural-peritoneal shunt, a long-term ambulatory or indwelling pleural catheter for drainage, and open pleurectomy. Each of these procedures can successfully relieve dyspnea, but they are all associated with potential complications. When the expected survival is short, less invasive procedures should be considered (e.g., repeated thoracocentesis to relieve the symptoms) [7,8,39].

For patients with MPE and an anticipated survival time of 6 months, treatment with long-term indwelling pleural catheters is comparable to treatment with talc pleurodesis [40,41]. There are no robust data to support which of these two treatments is more effective at palliating symptoms and improving quality of life [40-43].

### Study limitations

Our study has a few limitations as follows. Our data originated from a single referral center, the study was retrospective, there was a small number of patients in some categories, and there was a lack of patients with mesothelioma in the cohort. The absence of patients with mesothelioma in our cohort is probably owing to the fact that the incidence of this cancer is low in Brazil: 221 cases were reported from 2000 to 2011 [44].

## Conclusions

This study shows that ECOG PS is an independent predictor of survival in patients with MPE at the time of the initial diagnosis. ECOG PS is a significant prognostic factor that can help physicians to select patients for appropriate palliative treatment of this syndrome. However, more studies are needed to conclude that one single factor can be a predictor of survival. Moreover, these studies will probably need to be confirmed by prospective studies to determine the best selection of treatment for providing the best quality of care.
